# Disorders of sex development in Wolf–Hirschhorn syndrome: a genotype–phenotype correlation and *MSX1* as candidate gene

**DOI:** 10.1186/s13039-021-00531-8

**Published:** 2021-02-24

**Authors:** Khouloud Rjiba, Hédia Ayech, Olfa Kraiem, Wafa Slimani, Afef Jelloul, Imen Ben Hadj Hmida, Nabiha Mahdhaoui, Ali Saad, Soumaya Mougou-Zerelli

**Affiliations:** 1 Laboratory of Human Cytogenetics, Molecular Genetics and Biology of Reproduction, Farhat Hached University Teaching Hospital, Sousse, Tunisia; 2 Higher Institute of Biotechnology, Monastir University, Monastir, Tunisia; 3 Unité de Services Communs en Génétique Humaine, Faculté de Médecine de Sousse, Université de Sousse, Sousse, Tunisia; 4 Pediatric Department, Farhat Hached University Teaching Hospital, Sousse, Tunisia; 5 Pediatric Department, Regional Hospital, Kairouan, Tunisia

**Keywords:** Hypospadias, Array CGH, FISH, Wolf–Hirschhorn syndrome, *MSX1*gene

## Abstract

**Background:**

Wolf–Hirschhorn (WHS) is a set of congenital physical anomalies and mental retardation associated with a partial deletion of the short arm of chromosome 4. To establish a genotype–phenotype correlation; we carried out a molecular cytogenetic analysis on two Tunisian WHS patients. Patient 1 was a boy of 1-year-old, presented a typical WHS phenotype while patient 2, is a boy of 2 days presented an hypospadias, a micropenis and a cryptorchidie in addition to the typical WHS phenotype. Both the array comparative genomic hybridization and fluorescence in situ hybridization techniques were used.

**Results:**

Results of the analysis showed that patient 2 had a greater deletion size (4.8 Mb) of chromosome 4 than patient 1 (3.4 Mb). Here, we notice that the larger the deletion, the more genes are likely to be involved, and the more severe the phenotype is likely to be. If we analyze the uncommon deleted region between patient1 and patient 2 we found that the Muscle Segment Homeobox (***MSX1***) gene is included in this region. ***MSX1*** is a critical transcriptional repressor factor, expressed in the ventral side of the developing anterior pituitary and implicated in gonadotrope differentiation. *Msx1* acts as a negative regulatory pituitary development by repressing the gonadotropin releasing hormone (***GnRH***) genes during embryogenesis. We hypothesized that the deletion of ***MSX1*** in our patient may deregulate the androgen synthesis*.*

**Conclusion:**

Based on the ***MSX1*** gene function, its absence might be indirectly responsible for the hypospadias phenotype by contributing to the spatiotemporal regulation of ***GnRH*** transcription during development.

## Background

Over the past decade, advanced molecular cytogenetic analysis such as array CGH has made valuable contributions to the knowledge and refinement of several chromosomal regions involved in birth defects and has led to the emergence of several well-established chromosomal syndromes. Among these syndromes, chromosome 4p16.3 deletion [OMIM#194190] is a contiguous gene deletion syndrome resulting in several clinical features, including growth and mental retardation, microcephaly, seizures, “Greek helmet” facies, and major malformations such as cleft lip and/or palate (CL/P), coloboma of the eye, congenital heart defects (CHD) and dental anomalies (oligodontia) [1, 2]. The WHS syndrome was first described by Hirschhorn and Cooper in a preliminary report in 1961 and later formalized with back-to-back publications by Wolf et al., and Hirschhorn et al., in Humangenetik in 1965 [3]. Its frequency ranges from 1 case per 50,000 births to 1 case per 20,000 births, occurring more frequently in females with a male to female ratio of 1:2 [4]. Several literature reports point to the great variability of the WHS phenotype, depending mostly on the variability of the underlying genomic defect based on different size deletions [5, 6]. Hence, previous studies of 4p16.3 deletion focused largely on postnatal growth delay, CHD, and oligodontia. However, hypospadias has not been lighted in the phenotype. In this paper, we report on an additional case of a 4p16.3 deletion associated with hypospadias, micropenis, dysmorphic features, microcephaly, heart disorder, and Platine crack. Here, by reviewing the literature, we emphasize Disorders of sex Development (DSD) traits in the phenotype and suggest a candidate gene.

## Results

**Fig. 1:**
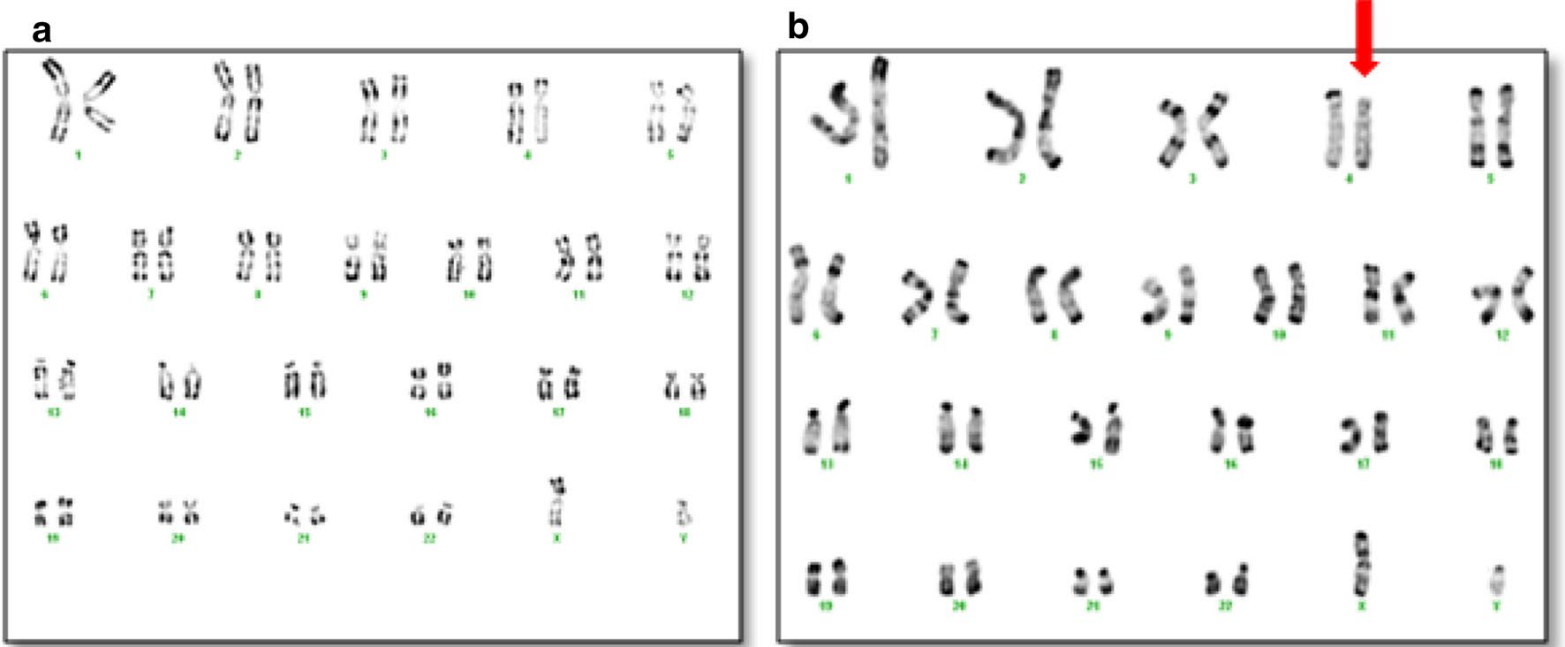
Karyotypes of both patients carrying the deletion at the chromosome 4p16 in the patient 2 (**b**) and the absence of the deletion in the patient 1 (**a**). The arrow shows the partial deletion 4p

**Fig. 2:**
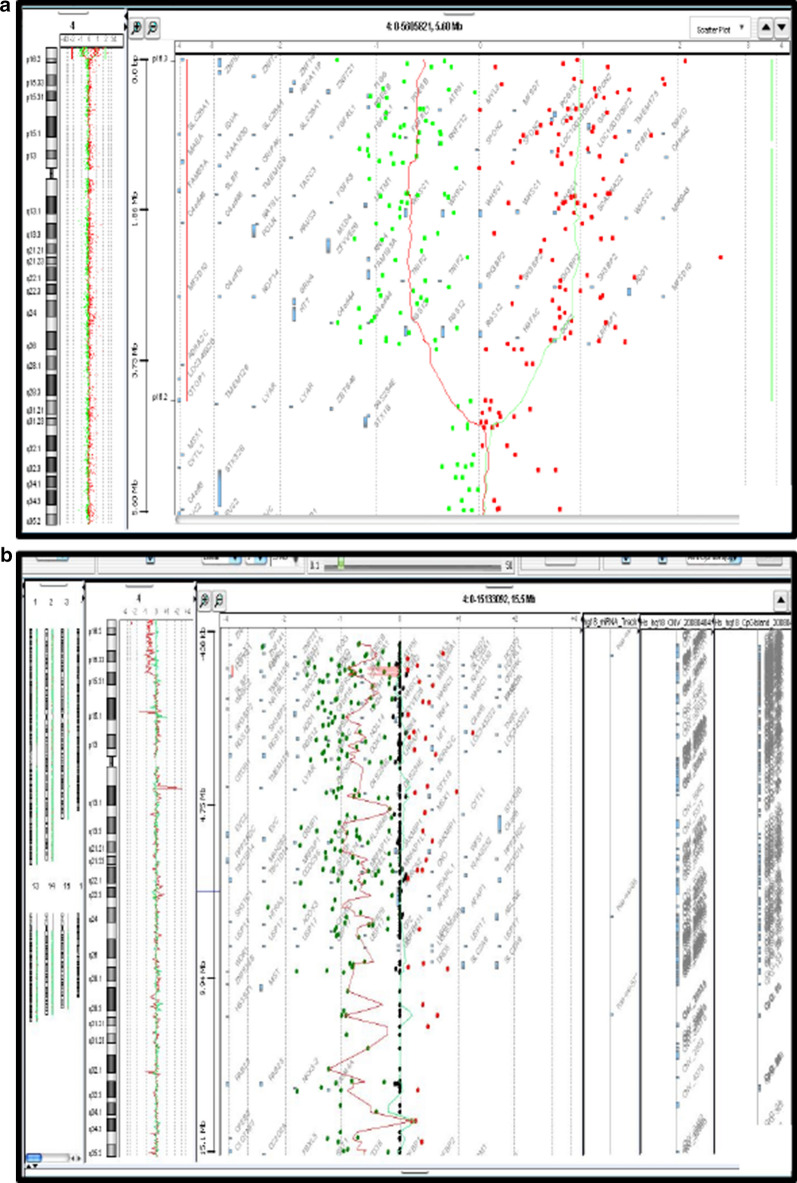
44,0000 Agilent Technologies oligonucleotides array profiles of both patients showing: **a** deletion of at least 3.4Mb in patient 1. **b** deletion of at least 4.8 Mb in patient 2

The chromosomal analysis of the first patient indicated a normal male karyotype 46, XY in all metaphases (Fig. 1a). Array CGH analysis revealed partial 4p deletion encompassing at least 3.4 Mb ranging from nucleotides 72,447 to 3,519,927 according to the Human reference genome hg18,46,XY.arr[hg18]4p16.3 (72,447_3,519,927) ×1 dn (Fig. 2a).

For the second patient, conventional Karyotype revealed a male karyotype with a terminal deletion of the short arm of chromosome 4;46,XY,del(4)(p16.3) (Fig. 1b). Parents’ R-banded karyotype from peripheral blood didn’t reveal any chromosomal anomalies in the resolution limit of banding detection. Array CGH characterized this deletion encompassing at least 4.8 Mb extending from nucleotides 62,447 to 19,065,971, according to the Human reference genome hg18,46,XY.arr[hg18]4p16.3(62,447–19,065,971) ×1 dn (Fig. 2b).

**Fig. 3:**
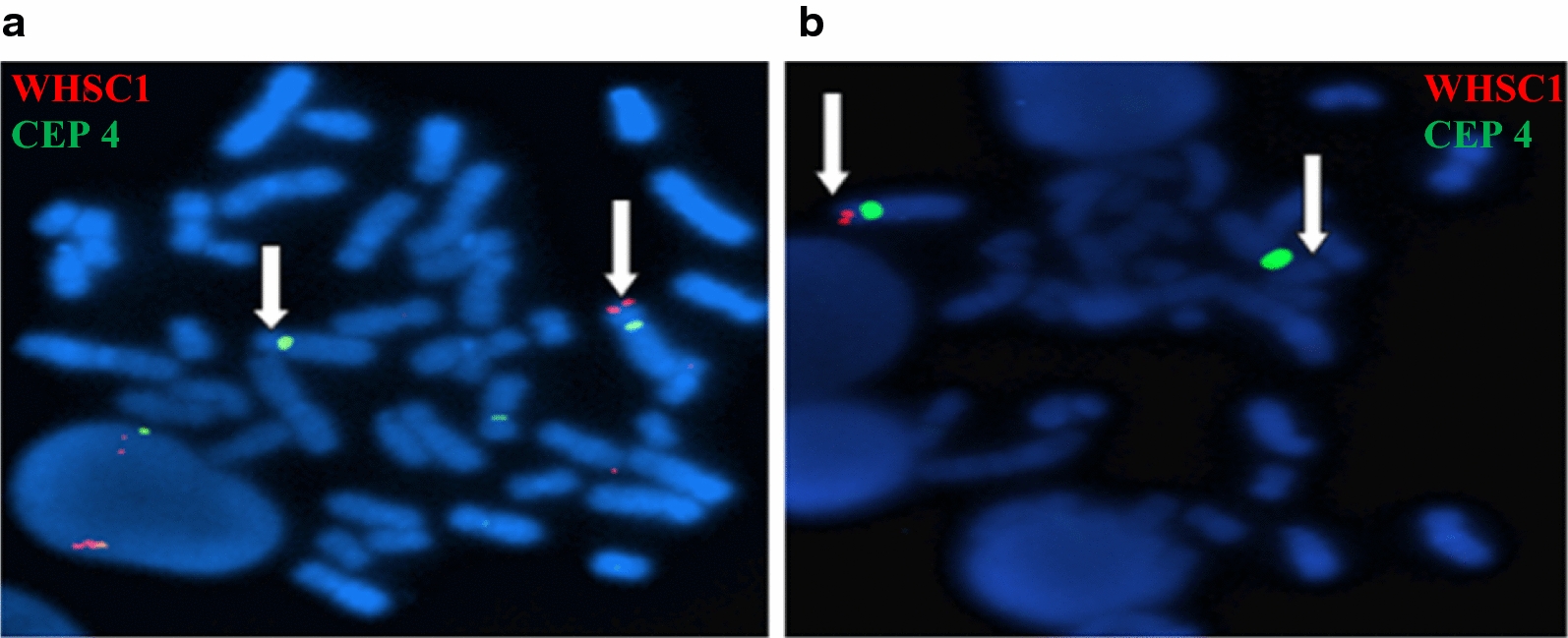
FISH analysis with commercial probes (Vysis Wolf–Hirschhorn Region Probe—LSI WHS Spectrum Red/CEP 4 Spectrum Green). the arrow showed the partial deletion of chromosome 4 in patient 1 (**a**) and patient 2 (**b**)

Then, FISH assay confirmed the chromosomal rearrangement by showing a partial deletion on chromosome 4, in both patients, using Kreatech dual colour probes (Fig. 3a, b).

## Discussion

The Wolf–Hirschhorn syndrome (WHS) is the first example of a human chromosomal deletion syndrome, described as a pathogenetic syndrome. It is usually caused by the deletion of the sub-telomeric short arm of chromosome 4 [1].

The spectrum and severity of WHS clinical features typically correlate with the deletion size [2–5]. This report focuses on the DSD as particular features in genotype–phenotype correlation analysis of WHS patients on two Tunisian patients and proposes a candidate gene to this developmental disorder.

The main characteristic of WHS is the typical face, usually referred to as a “Greek warrior helmet face”. Previous studies suggest a critical region that, when deleted, causes the recognizable syndrome. It has been narrowed to a 165 kb, about 1.9 Mb from the 4p telomere, and includes two purported regions, called WHSCR1 and WHSCR2 [6, 7]. So far, advanced molecular techniques such as FISH and array CGH provided the possibility of detecting smaller deletions with less evident phenotypes.

Our study focuses on variable features in two 4p deletions cases. Molecular and conventional cytogenetic analysis, showed a partial loss of 4p with different breakpoints and different size deletions. Both patients are clinically suspected to have WHS. Patient 1 has specific dysmorphic features, a severe psychomotor delay, failure to thrive and microcephaly. His clinical profile is suggestive of a mild form. However, patient 2 has a polymalformative syndrome including dysmorphic features: a microcephaly, a megalocornea, an hypertelorism, a microretrognatism and a growth delay. He presents a heart defect, a Palatine crack and a cryptorchidism, micropenis and hypospadias, all the three last features are suggestive of sex development disorder in atypical WHS case. Array-CGH exploration characterized the 4p loss of 3.4 Mb in the first patient and of 4.8 Mb in the second. We suggest that different deletion sizes and the variability of the involved genes could play an important role in the complex phenotype of WHS in each patient (Table 1).

**Table 1:** Comparison of the phenotypic features in patients with 4p deletion

Paper	Flipsen-ten Berg et al. (2007) [37]	Chen et al. (2011) [38]	Sifakis et al. (2012) [39]	Malvestiti et al. (2013) [40]	Venegas-Vega et al. (2013) [41]	Present study	
Patient reference	Patient 1	Patient 1	Patient 1	Patient 1	Patient 1	Patient 1	Patient 2
Size of deletion, Mb	8.3	6.5	14.7	6.29	6.48	3.4	4.8
Deleted region	4pter-p16.1	4p16.3-p16.1	4p15.33-pter	4p16.3	4p16.1-p16.3	4p16.3	4p15.3–16.2
Age at diagnosis, years	1 and 2 months	Prenatal	Prenatal	Prenatal	9 and 9 months	1	2 days
Gender	M	M	M	M	M	M	M
Cranio-facial dismorphism	−	+	+	+	+	+	+
Growth retardation	+	+	+	+	NA	+	+
Microcephaly	+	NA	NA	NA	+	+	+
Neurological features hypotonia	+	+	NA	+	+	+	−
Hypertelorism	+	+	NA	NA	NA	−	+
Delayed mental development	+	+	NA	NA	+	−	−
Delayed motor development	+	NA	NA	NA	+	+	−
Hypospadias	+	+	NA	NA	NA	−	+
Cryptorchidism	+	NA	NA	+	NA	−	+

+, present, −, absent; NA, not available

In order to understand the genotype–phenotype correlation in both cases, we focus on the uncommon deleted region. However, the common deleted region, in both cases, involves three important genes implicated in the development of the main features of WHS. These genes are ***WHSC1***, ***FGFRL1***, and ***LETM1***. The Wolf–Hirschhorn syndrome candidate 1 gene (***WHSC1***), also known as ***NSD2*** (nuclear receptor SET domain containing) and ***MMSET*** (multiple myeloma SET domain containing) [OMIM#602952], is located in the WHSCR1 region and its loss is believed to be responsible for several features of the syndrome. It encodes a putative histone methyltransferase, and the resulting protein has several domains [8, 9].

The molecular features of ***WHSC1*** suggest different functions such as a chromatin-remodeling enzyme function since its SET domains act as histone methylase. A deficiency could then deregulate multiple genes expression leading to a pleiotropic effect [10]. Recently, it has been proposed that deficiency in ***WHSC1*** gene leads to defects in the DNA damage response as seen in WHS patients. The ***WHSC1*** has been indeed localized at sites of DNA damage and replication stress and then is required for resistance to many DNA-damaging and replication stress-inducing agents [11, 12]. This function could then explain the neurological impairment in WHS. However, the hypothesis that typical WHS could be a single-gene disorder is unlikely. We think that the full WHS phenotype results from the haplo-insufficiency of several other candidate genes, especially those telomeric to ***WHSC1***. The best possible interpretation of the currently available data, in patients 1 and 2, is that ***WHSC1***, in combination with closely linked genes, are responsible for the core phenotypes.

Interestingly, the common deleted interval in both patients encompasses the Fibroblast Growth Factor Like-1 gene (***FGFRL1***) [OMIM#605830] considered as the most characterized gene in this region. Located on 4p16.3 outside and distal to the WHSCRs, the ***FGFRL1*** gene encodes a member of the fibroblast growth factor receptor family [8]. Recent studies suggest that ***FGFRL1*** represents a plausible second candidate gene for several other WHS features. Mouse models targeting ***FGFRL1*** present growth delay, craniofacial defects, skeletal anomalies and congenital heart defects features that are in complete accordance with WHS phenotype, mainly the craniofacial phenotype [6, 13, 14]. In the proximal side of ***WHSC1***, additional genes contributing to the core phenotypes may act to complete the pleiotropic WHS phenotype. Leucine zipper/EF-hand-containing transmembrane gene (***LETM1***) [OMIM#604407], an ubiquitous Ca2 + binding protein involved in Ca2 + homeostasis, is located at 1.8 Mb from the telomere. This gene has been suggested to cause seizures [7, 15] and seems to be the most likely candidate gene for epilepsy in WHS patients. Indeed, impaired Ca2 + homeostasis in nerve cells has been correlated with neurodegenerative disorders and seizures [16–19]. In the present study, while the ***LETM1*** gene is deleted in both cases only the first patient presents epilepsy. Elsewhere, it has been previously reported a WHS patient suffering from seizures with a 1.4 Mb terminal 4p deletion preserving ***LETM1*** gene [2]. In another study, six of eight subjects with terminal 4p deletions preserving ***LETM1*** had seizures, whereas seven of seven with small interstitial deletions including ***LETM1***, did not [7]. Taken together, it seems that ***LETM1*** haploin sufficiency contributes to seizure genesis but epileptic phenotype genesis appears to be questionable and not fully elucidated and another gene or genes could be incriminated. As advanced elsewhere C-Terminal-binding protein 1, a transcriptional co-repressor gene (***CTBP1***) [OMIM#602618], could be a good candidate for seizures/epilepsy in WHS [20, 21].

The Wolf–Hirschhorn syndrome candidate 2 (***WHSC2***) [OMIM#606026], encodes a subunit of the negative elongation factor complex, involved in mRNA processing and the cell cycle [22, 23]. This complex seems to induce promoter-proximal pause by inhibiting RNA polymerase II early progression during elongation, and consequently altering the expression of its target genes [24]. Recently, ***WHSC2*** has been implicated in the recruitment of Stem Loop Binding Protein (***SLBP***) [OMIM#602422] to the 3′ ends of histone pre-mRNAs [22]. Taken into account that the ***SLBP*** gene is included in the patients1 and 2 deletions, we suppose that haploinsufficiency of ***SLBP*** and/or ***WHSC2*** supply microcephaly, pre- and postnatal growth retardation, the core clinical features of WHS. Employing a unique panel of patient-derived cell lines with differently-sized 4p deletions, underlies novel cellular defects associated with WHS. It has been demonstrated that haploinsufficiency of ***SLBP*** and/or ***WHSC2*** contributes to delayed cell-cycle progression, impaired DNA replication and altered chromatine structure [25]. These results may explain the phenotype severity observed in the present patients too suggesting a functional relationship between both genes ***SLBP*** and ***WHSC2***, commonly haploinsufficient in WHS.

In addition, in the present study, we report on the deletion of Chromosome 4 Open Reading Frame 48 (***C4ORF48***) [OMIM#614690], a gene located in a 191.5-kb region and associated to WHS patients presenting microcephaly and growth retardation. Interestingly, expression of ***C4ORF48*** in different zones during cortical and cerebellar development, as well as in almost all cortical and subcortical regions of the adult mouse brain was proven [26]. This suggests a potential role of ***C4ORF48*** in the development of human cerebral and cerebellar structures, and plasticity function in adult brain neurons. It indicates also that ***C4ORF48*** hemizygosity might be partly involved in the WHS neurological aspects.

Otherwise, if we focus on the differential features and the non-overlapping region between the two patients 1 and 2 in the present report, we notice that the different genes involved may explain the presence of a sex development disorder in patient 2. A deep analysis of this region underlies a deletion of the ***MSX1*** gene [OMIM#142983] at 4.9 Mb from the telomere. As previously seen monosomy of ***MSX1*** was linked to the oligodontia observed in some WHS patients suggesting that selective tooth agenesis is a common phenotype in Wolf–Hirschhorn syndrome [27, 28]. It could be considered then as an obvious candidate gene for the cranio-facial structures and the anterior forebrain development [29]. ***MSX1*** has been reported also as a transcriptional repressor of GnRH promoter activity that is expressed in the ventral side of the developing anterior pituitary. It is regulated by Bone Morphogenetic Protein (BMP), and implicated in gonadotropin neurons differentiation [30, 31].

Interestingly, some other studies mapped the critical region for hypospadias in WHS syndrome between 3 Mb and 4.0 Mb [2, 32]. Taken into account the deleted region of the second patient in the present report, it is possible that an haploinsufficiency of the ***MSX1*** gene could explain the hypospadias phenotype.

As known, proper sexual maturation depends upon the correct function of the hypothalamic–pituitary–gonadal axis, initiated by a critical population of GnRH neurons [33] and then, by binding to the consensus homeodomain repeats (ATTA) in the enhancer and promoter, ***MSX1*** could repress GnRH promoter activity and consequently participate in the regulation of ***GnRH*** gene expression network [32].

Thus, it may deregulate the androgen synthesis; which may lead to hypospadias during an embryogenesis critical phase. Indeed, recently, ***MSX1*** has been proposed as a candidate gene for hypogonadism based on its function in the gonadotropic axis [34].

Curiously, the ***MSX1*** deletion in the second patient is associated with hypospadias without the expected oligodontia. Here we could explain these controversies by variable expressivity or incomplete penetrance. Several mutations in the homeodomain of ***MSX1*** are associated to tooth agenesis or orofacial clefts [35].

But to the best of our knowledge, no reported ***MSX1*** gene mutations have been associated to DSD. Here, again we underlie the acting network in a multiple genes deleted syndrome as WHS.

In summary, we suggest ***MSX1*** gene as an intriguing candidate gene for contribution to the hypogonadal phenotype. Functional studies for ***MSX1*** gene should be considered to more understand its implication in the development of oligodontia and hypospadias.

Here we emphasize the phenotype-genotype correlation studies, which are considered as the core, the beginning, and the end of gene analysis. The use of a combined approach conventional cytogenetic and, chromosomal array associated with a deep analysis of a molecular and functional gene studies are necessary.

Based on the genome system theory, the correlation between the size of deletion and severity of diseases might be explained by the alteration of karyotype coding based on a “system inheritance” which consider genes and the genomic topology within the three-dimensional nucleus configuration [36]. Based on the new emergent genome, we have posited that chromosomal rearrangements with different size as seen here can reorganize different genomic information’s leading to an abnormal development and then pathologic phenotypes.

Taking all these facts into consideration, functional studies or more sophisticated technologies such as Hi-C technologies are highly recommended to better characterize the genetic interactions following 4p deletion. It is likely that more patients with WHS will present hypogonadism and therefore precise personal medical care is required.

## Conclusion

In conclusion, WHS is a multigenic syndrome with a spectrum of phenotypic features, from very subtle and mild to a wide range of severe aberrations. Array CGH allowed us to better identify the breakpoints and genes likely to be involved in the WHS syndrome. Therefore, our work highlights new candidate genes such as ***MSX1*** gene likely responsible of hypogonadism in WHS. It allows establishing a specific genotype–phenotype correlation and underlining the new genomic topology tools as relevant to understand the role of the different 4p genes in the WHS development.

## Methods

### Karyotype

Conventional cytogenetic analysis was performed on the peripheral blood lymphocytes according to standard procedures. Chromosome analysis was carried out applying R-banding at a 450 band resolution according to ISCN 2016 in both patients and their parents. Metaphase chromosome spreads were prepared from phytohemagglutinin—stimulated peripheral blood lymphocytes-based on standard protocol. Cell cultures were incubated for 72 h. At least 20 mitoses were investigated for each sample using Cytovision® Karyotyping software version 4.0.

### Fluorescent in situ Hybridization (FISH)

FISH was performed on blood lymphocytes blocked on metaphases of each patient, according to the standard protocol. FISH followed manufacturer’s instructions, using probes for chromosome 4 (Vysis® Wolf Hirschhorn Region probe-LSI WHS (Red) and CEP 4 (Green) (Vysis, Abbott Laboratories, IL, USA). Probes were applied to metaphase slides and therefore co-denaturized for 7 min at 75 °C. After overnight hybridization at 37 °C, the slides were washed for 5 min in de 2XSSC/ NP40(Vysis, Illinois, Unites States) at 75 °C. Chromosomes were mounted with a 4,6 diamino-2-phenylindole and analyzed using an Axioskop Zeiss® fluorescent microscope.

### Array CGH

Array comparative genomic hybridization (array CGH) was performed with Agilent Human Genome array CGH Kit 44 K, for both patients, according to the manufacturer’s instructions (Feature Extraction 9.1, CGH Analytics 4.5, and Santa Clara, California, United States). The coverage of the human genome was made with an average spatial resolution of 75,000 pair bases. A copy number variation was noted when at least three contiguous oligonucleotides presented an abnormal ratio greater than + 0.58 or lower than − 0.75.

An in-silico analysis of the unbalanced region indicated by the analysis was made using UCSC Genome Browser (http://genome.ucsc.edu/), the Online Mendelian Inheritance in Man database (OMIM: https://omim.org/) and the Database of Genomic Variants (DGV: http://dgv.tcag.ca/dgv/app/ home).

### Clinical description

#### Patient 1

**Fig. 4:**
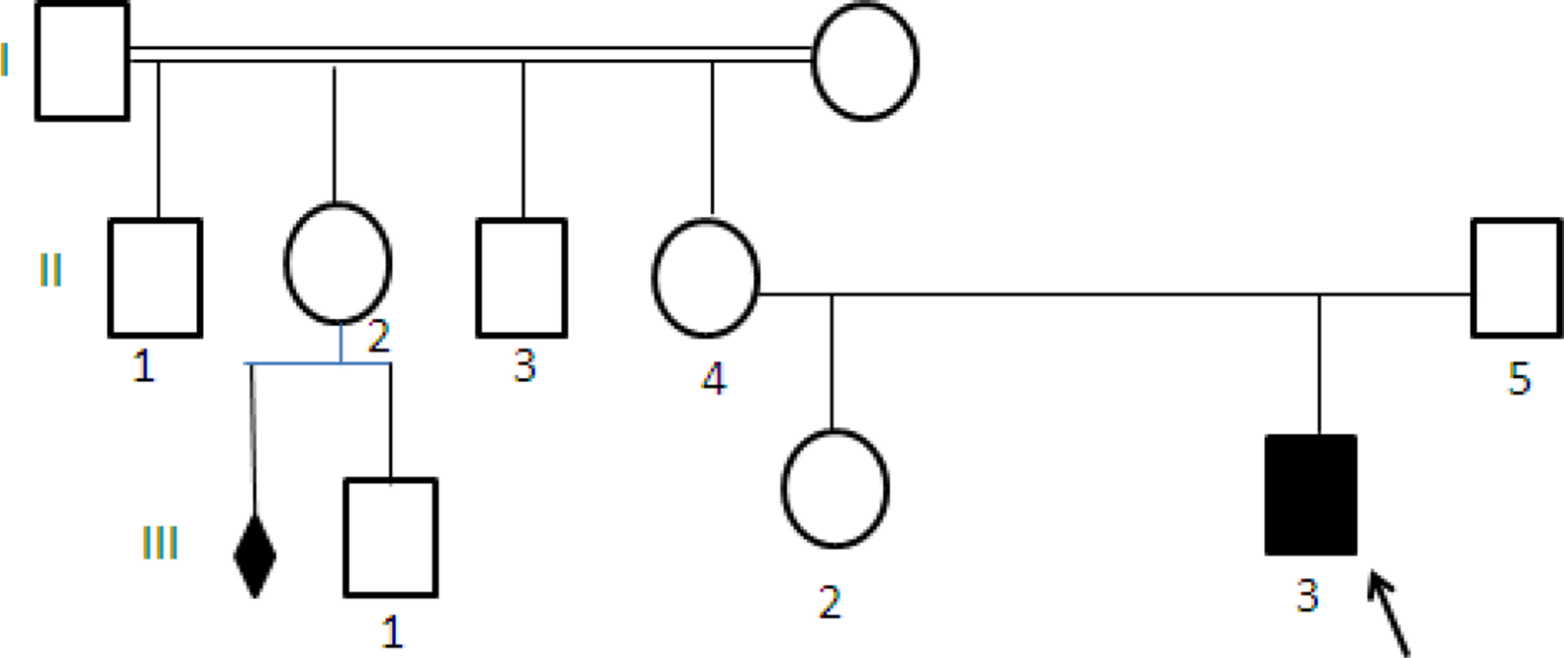
Pedigree of the family of patient 1

Patient 1(III3) is a 1-year-old boy, suffering from epilepsy since the age of 17 months. In addition he presents a specific dysmorphic features, a psychomotor development delay, growth retardation (weight (− 3.8) SD; size (− 5.3) SD) and a microcephaly. It is noteworthy that the patient had a maternal aunt with malformation syndrome (Fig. 4).

#### Patient 2

**Fig. 5:**
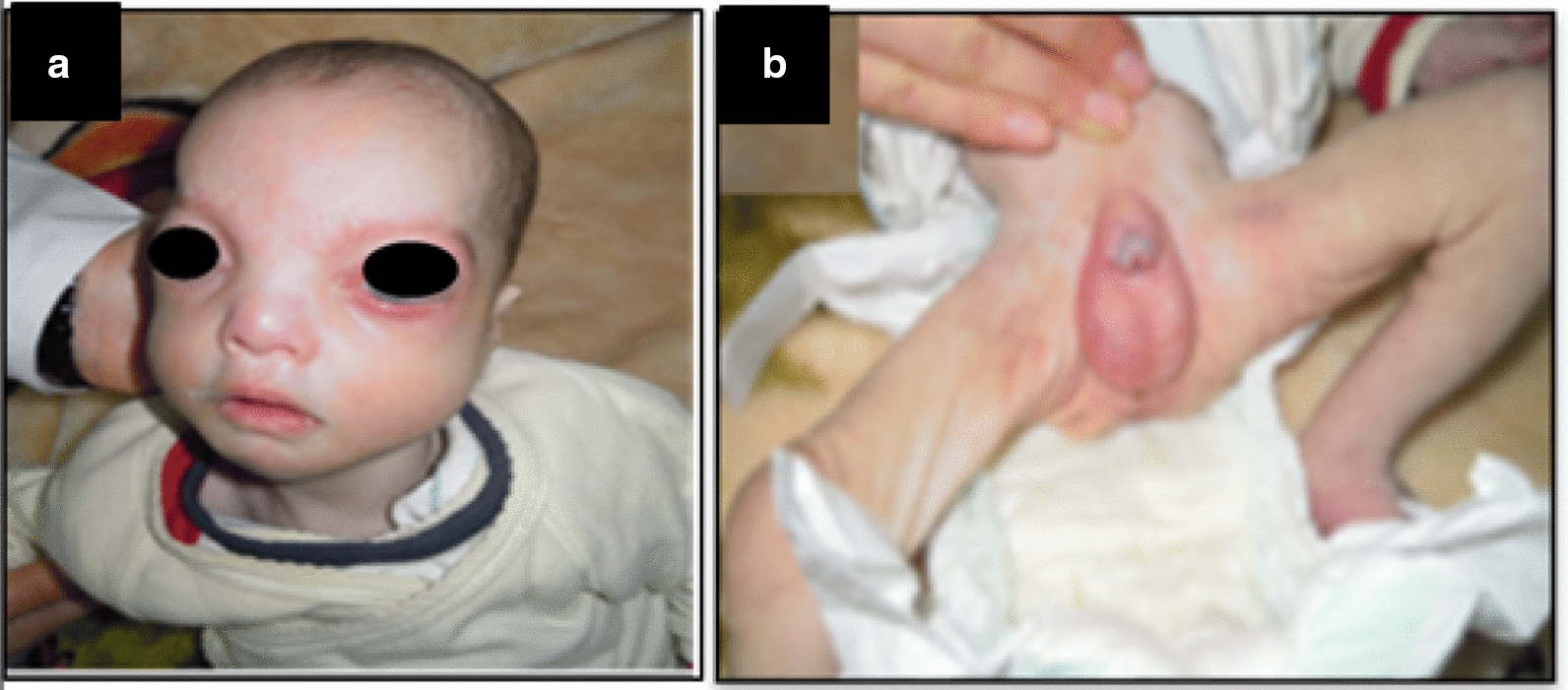
Photographs of profile picture (**a**) and external genital organs (**b**) of patient 2

Patient 2 is a newborn boy aged 2 days. At physical examination, he presents a polymalformative syndrome, suggestive of Wolf Hirshhorn syndrome, including dysmorphic features, microcephaly, a megalocornea, a hypertelorism, a microretrogandism, a heart disorder and a Platine crack. Furthermore, he had a disorder of sexual development type cryptorchidie, micropenis and hypospadias (Fig. 5).

## Data Availability

Data and materials are available from the corresponding author and available upon request.

## Abbreviations

Array CGH: Array comparative genomic hybridization
CHD: Congenital heart defect
DAPI: Diamino-2-phenylindole
DNA: Desoxyribonucleic acid
DSD: Disorders of sex development
FGFRL1: Fibroblast growth factor like-1 gene
FISH: Fluorescence in situ hybridization
GnRH: Gonadotropin releasing hormone
ISCN: International System for Human Cytogenetic Nomenclature
LETM1: Leucine zipper/EF-hand-containing transmembrane gene
MMSET: Multiple myeloma SET domain containing
MSX1: Muscle Segment Homeobox
NSD2: Nuclear receptor SET domain containing
OMIM: Online Mendelian Inheritance in man
SLBP: Stem Loop Binding Protein
WHS: Wolf–Hirschhorn syndrome
WHSCR1: Wolf–Hirschhorn syndrome region 1
WHSR2: Wolf–Hirschhorn syndrome region 2
WHSC1: Wolf–Hirschhorn syndrome candidate 1 gene
WHSC2: Wolf–Hirschhorn syndrome candidate 2 gene
